# Pilot Study and Preliminary Results of Biodegradable Intramedullary Nailing of Forearm Fractures in Children

**DOI:** 10.3390/children9050754

**Published:** 2022-05-20

**Authors:** Christoph Roeder, Cristina Alves, Andreas Balslev-Clausen, Federico Canavese, Erol Gercek, Tamás Kassai, Thomas Klestil, Louise Klingenberg, Nicolas Lutz, Marcell Varga, Gergo Jozsa, Annelie Weinberg, Ludger Tüshaus

**Affiliations:** 1 Department for Health Sciences, Medicine and Research, Danube University Krems, 3500 Krems, Austria; thomas.klestil@moedling.lknoe.at; 2 Department of Orthopedics and Trauma Surgery, Landesklinikum Baden/Moedling, 2340 Moedling, Austria; 3 Department of Pediatric Orthopaedics, Hospital Pediátrico de Coimbra, 3000-602 Coimbra, Portugal; 6443@chuc.min-saude.pt; 4 Department of Orthopedics, Hvidovre Hospital, 2650 Hvidovre, Denmark; andreas.peter.balslev-clausen@regionh.dk (A.B.-C.); louise.klingenberg.03@regionen.dk (L.K.); 5 Department of Pediatric Orthopedic Surgery, Jeanne de Flandre Hospital, 59000 Lille, France; federico.canavese@chru-lille.fr; 6 Department of Orthopedics and Trauma Surgery, Helios University Hospital Wuppertal, 42283 Wuppertal, Germany; erol.gercek@web.de; 7 Department of Pediatric Trauma Surgery, Dr. Manninger Jenő Baleseti Központ, 1081 Budapest, Hungary; kassai.tamas@obsi.hu (T.K.); drvmarcell@gmail.com (M.V.); 8 Service de Chirurgie de l’enfant et de l’adolescent, Centre Hospitalier Universitaire Vaudois, 1011 Lausanne, Switzerland; nicolas.lutz@chuv.ch; 9 Medical Centre, Department of Paediatrics, Division of Paediatric Surgery, Traumatology, Urology and Paediatric Otolaryngology, UP Clinical Centre, 7623 Pécs, Hungary; dr.jozsa.gergo@gmail.com; 10 Department of Orthopedics and Trauma, Medical University of Graz, 8036 Graz, Austria; anneliemartina.weinberg@medunigraz.at; 11 Department of Pediatric Surgery, University Medical Center Schleswig-Holstein, Campus Lübeck, 23538 Lübeck, Germany; ludger.tueshaus@uksh.de

**Keywords:** fracture, children, forearm, intramedullary nailing, bioabsorbable

## Abstract

(1) Background: Diaphyseal forearm fractures are a common injury in children and adolescents. When operative treatment is needed, elastic stable intramedullary nailing (ESIN) is the most common surgical procedure. Although there is no clear evidence, hardware removal after fracture healing is performed in many patients. Often, the primary minimal invasive incision needs to be widened during implant removal. In order to decrease the burden of care of pediatric fractures, significant efforts were made to develop biodegradable implants, which make hardware removal unnecessary. Our study will conduct an observational trial on the clinical use of the Activa IM-Nail™ in forearm fractures in children between 3 and 13 years of age. The objective of this trial is to evaluate the risks and benefits of the Activa IM-Nail™. Among other objectives, the rate of refracture will be determined. (2) Methods: An international Europe-based, multicenter, prospective, single-arm, open-label study will be performed to ascertain the rate of refracture and to determine the subjective benefits of Activa IM-Nail™ for patients, parents and other caregivers. The study will include clinical follow-up including early post-operative complication, radiographs until bony healing and an additional follow-up after 1 year. At this stage, preliminary results and early complications on 76 patients are analyzed in this study and presented. (3) Results: As of April 2022, 76 patients were enrolled as per study protocol. There were 31 girls (40.8%) and 45 boys (59.2%). The mean age at the time of inclusion was 8.9 years (±2.4 years). The mean operation time was 58.9 ± 22.9 min (range, 15–119 min). The mean follow-up time was 8.9 ± 5.1 months (range, 0.2–18.6). Up to now, one refracture has occurred in one child falling from a height of about one meter 7 months after index surgery (1/76; 1.3%). (4) Conclusion: The research project assesses the safety and effectiveness of Activa IM-Nails™ as part of the surgical treatment of dislocated forearm fractures in children in the context of a PMCF study. The use of Activa IM-Nails™ with regard to various objectives, including postoperative complications and refracture rate, seems to be equal to the standard titan ESIN procedure compared to the literature. Preliminary results are encouraging and are made available.

## 1. Introduction

Forearm fractures are among the most frequent fractures in children and adolescents [1,2]. Treatment is heterogeneous and varies according to fracture location, severity of displacement, patient’s age and surgeon’s preference. Conservative treatment is mostly indicated in patients with enough growth remaining and in those with mildly displaced or un-displaced fractures. On the other hand, surgery is recommended in displaced or unstable fractures, where remodeling potential is low—especially in the midshaft and proximal—and in older patients with lower residual growth potential. Elastic stable intramedullary nailing (ESIN), Kirschner wire fixation, external fixation, plating or hybrid fixation are the available surgical options. Surgical treatment can prevent axial deformity which may affect function, particularly pronation and supination.

Although evidence-based guidelines are missing [3], many surgeons recommend hardware removal after fracture healing [4,5] even though complications related to it can occur in up to 10% of cases [3]. In addition, economic implications are not negligible, due to the costs of hospital stays and working time lost by parents or guardians.

Parents and surgeons are also concerned about potential long-term consequences of retained metallic implants, as they can provide a stable host surface for intraoperatively derived bacteria and can release metallic ions, though the long-term effects of these are not yet fully known [6]. Metallic hardware can also be a cause of pain, irritation, pseudobursitis and stress-shielding. In addition to those long-term consequences, the removal surgery can sometimes be more invasive than the primary operation, as the incision needs to be widened in many cases. Removal-associated complications after ESIN osteosynthesis are reported by Lieber et al. [7].

To counteract all these issues, considerable effort was put into developing biodegradable materials for osteosynthesis [8] that allow fracture stabilization and do not require implant removal. These implants also offer the advantage of gradual load transfer to the healing tissue and reduce the risk of stress-shielding, as reported by several authors [9,10,11,12]. In particular, Gortzak et al. described the use of bioabsorbable fixation for pediatric olecranon fractures [8], Partio et al. used bioabsorbable hardware for the fixation of subtalar extra-articular arthrodesis in children [9], while Hope et al. compared self-reinforced absorbable rods with metallic fixation in children with elbow fractures [8].

The idea of treating forearm fractures with bioabsorbable intramedullary nails was introduced in the early 1990s by Finnish surgeons. Sinikumpu et al. first reported on the clinical use of polymers polyglycolide (PGA) and polylactide (PLA) and their copolymers (PLGA and PLDLA). A preliminary technical report on the use of PLGA for intramedullary nails in pediatric forearm fractures was published in 2013 [10]. Later, the randomized controlled trial by Korhonen et al. compared titanium elastic nails with the biodegradable Activa IM-Nail™ (Bioretec Ltd., Tampere, Finland) [11]. The study reported no difference in range of motion or fracture healing between the two groups, but two refractures occurred among patients treated by bioabsorbable nails. Refracture is a known complication of forearm fractures and varies between 1.1% and 16.7% during the first postoperative year [12,13,14,15,16,17,18,19,20,21,22,23,24]. According to Amerstorfer et al., the greatest risk of this appears to be in the first eight months after the injury [25]. The one year cut off is based on adult studies, while growing bone seems to be less vulnerable 8 months after injury [12,25]. Nevertheless, further research is required to evaluate the safety of the Activa IM-Nail™.

The aim of this pilot study is to present the background, the rationale and the methodology of a large multicenter study evaluating the rate of refracture and to determine the subjective benefits for the patient and the parents. The secondary aim is to present the preliminary data on the first 76 patients enrolled in the pilot study, so far.

## 2. Materials and Methods

### 2.1. Study Design, Background and Rationale

The pilot study, based on a prospective, multicenter, observational, clinical trial within the European Union, will assess the refracture rate of patients treated with Activa IM-Nail™ and casting.

**Figure 1:**
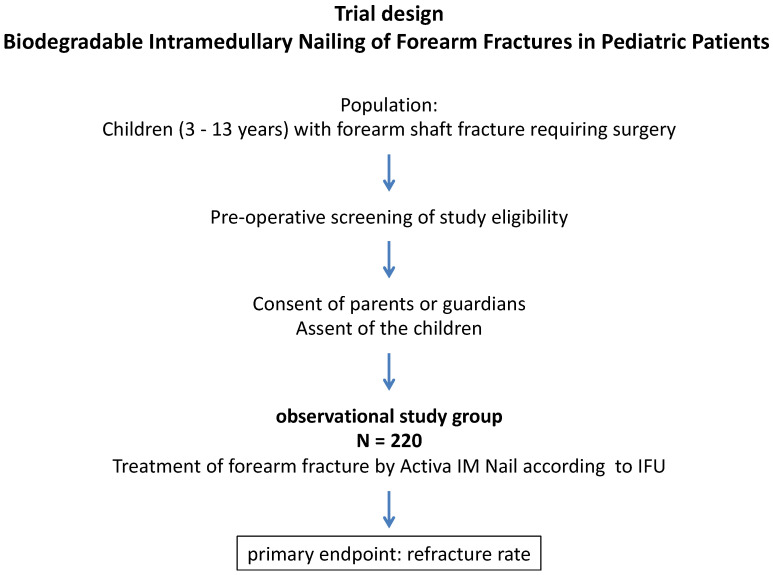
Flow chart of the trial design.

The protocol conforms to the Standard Protocol Items: Recommendations for Interventional Trials (SPIRIT) guidelines (see additional file 1). Figure 1 shows a flow chart of the trial design. Study sites are listed in Table 1.

### 2.2. Consent

Before any protocol-specific procedure is performed, the patient and the patient’s legal guardian will have to sign and date the informed consent. During the consent procedure, the patient and the patient’s legal guardian will be informed of all elements of the postmarket clinical follow-up (PMCF) study. Sufficient time will be given to the child and the patient’s legal representative so that questions can be asked and a voluntary and well-informed decision can be made. To be enrolled in the study, all patients must meet the inclusion criteria.

The PMCF study participation is voluntary, and at any time the patient may terminate participation and withdraw from the study. If the investigator considers that further participation in the study is not in the best interest of the child, the patient may also be withdrawn from the study at any time. Regardless of the reason for withdrawal, withdrawn children will not be replaced. Data provided up to the time of withdrawal will be collected and reported. This will be documented in the patient’s original records and in the electronic case report form (eCRF).

### 2.3. Participants

All pediatric patients between 3 and 13 years, admitted to one of the study sites because of a forearm fracture requiring surgical stabilization will be assessed for eligibility. The diagnosis will be verified by radiographs in two planes. The patient’s legal guardian will be introduced to the study, asked to participate and to sign the written informed consent form. Inclusion and exclusion criteria are listed as follows.

### 2.4. Inclusion Criteria

- Diaphyseal forearm fractures (radius or ulna or both).
- Surgical stabilization is required.
- Time between injury and operative treatment not exceeding 14 days.
- Patients from 3 years to 13 years (chronological age).
- The patient and the patient’s legal guardian have signed the informed consent form and are willing to participate in all follow-up visits.

### 2.5. Exclusion Criteria

- Spiroid fractures, multifragmentary fractures.
- Metaphyseal and epiphyseal fractures.
- Fractures where internal fixation is otherwise contraindicated, e.g., active or potential infection, pathological fracture (malignancy) or when a patient’s cooperation cannot be guaranteed.

Justification: The inclusion and exclusion criteria are according to Activa IM-Nail™’s indication and instruction for use.

### 2.6. Participant Withdrawal Criteria

Study participation may be terminated at any time. This may occur for the following reasons:

- at the request of the patient or his or her legal representative.
- on the part of the study by the investigator if it is considered that continued study participation is not in the best interest of the child.

(See also Section 2.2).

### 2.7. Participant Timeline

The flowchart for the study timeline is shown in Figure 1.

The specific time points for enrollment, interventions, clinical examinations, and follow up visits are listed in Table 2.

### 2.8. Sample Size

The primary aim of the pilot study is to evaluate the re-fracture rate in patients treated with Activa IM-Nail™. Based on a review in literature, the average rate of re-fracture following ESIN treatment is 4.9% (range: 1.1% to 16.7%). This was based on a total of 1952 patients from 13 studies identified with a PubMed search [12,13,14,15,16,17,18,19,20,21,22,23,24]. A total of 96 refractures were reported in these studies, either with ESIN in situ or during follow up after hardware removal. The sample size of this PMCF study was based on the following assumptions: a refracture rate of 5% (similar to ESIN) and the desired precision of ±3% as expressed in the 95% confidence interval for the estimated rate. This resulted in the sample size of 203 patients with follow-up information available. With an assumption of 8% lost to follow up, we decided to include 220 children.

### 2.9. Allocation, Randomization and Blinding

Not applicable, as there is only one study group.

### 2.10. Intervention

**Figure 2:**
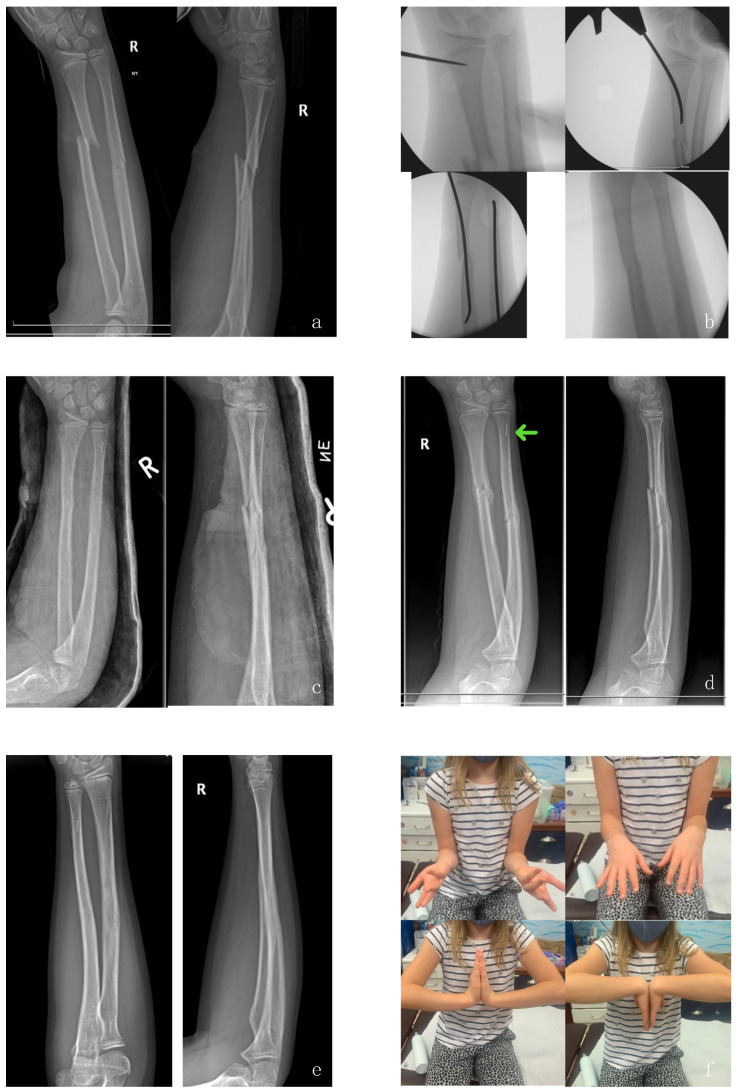
(**a**) Preoperative X-ray; (**b**) intraoperative; (**c**) one week; (**d**) six weeks (arrow marks the radiopaque tip of the implant); (**e**) one year; (**f**) range of motion after one year.

The surgery will be performed by a pediatric surgeon. In surgery, standards of the hospital’s pre-operative care will be applied. The surgery follows the “Instructions for Use” (IFU) provided by Bioretec Ltd. and is similar to the technique of ESIN. A detailed description of the surgical procedure is available in chapter “additional files”. The radiograph in Figure 2 illustrates the placement of the devices after surgical intervention.

### 2.11. Outcomes

Radiological evaluation of the injury, fracture alignment, angulation and union, as well as functional evaluation of outcome and rate of complications, will be performed immediately and at 2 weeks, 3 months and 1 year. Additionally, some of the study sites will perform magnetic resonance imaging (MRI) scans after one and two years to assess the process of biodegradation of the implant and possibly occurring subclinical soft-tissue reactions.

#### 2.11.1. Definition of Fracture Healing

It was determined that bones should be radiologically considered healed if the bony overgrowth in at least 3 of 4 cortices at the fracture site in AP and lateral views is detected. New radiographs will be evaluated at each clinical visit for signs of fracture healing. All radiographs will be subsequently evaluated independently by two experienced pediatric traumatologists (CR and LT) for fracture healing as well.

#### 2.11.2. Primary Outcome Measures

The primary objective will be to assess clinical outcome by determining the refracture rate of all treated patients and the difference in re-fracture rate depending on the fracture type determined by X-ray (e.g., greenstick vs. non-greenstick fracture), patients’ age, body mass index (BMI), surgical technique, immobilization time and bony union formation.

#### 2.11.3. Secondary Outcome Measures

- At some of the study sites, MRI scans at one and two years after surgery will be performed to assess the amount of biodegradation and to detect soft-tissue reaction caused by the implant.
- Cost effectiveness of this treatment will be evaluated in a health technology assessment 1 year after surgery of the last patient included in the study.
- Evaluation of bony union depending on fracture type and immobilization time, return to daily activity, return to sport.
- Safety and performance of operative technique in Monteggia’s lesions.

### 2.12. Data Collection and Management

An eCRF was designed and it will be used for data collection and management. Entered data in the eCRF is pseudonymized and only the treating study site will be able to link the pseudonymized data to the respective patient.

After article publication of results, individual participant data that underline the results are shared after deidentification with investigators whose proposed use of the data has been approved by an independent review committee identified for this purpose. Proposals may be submitted after article publication to the corresponding author. Additionally, all final, anonymized datasets and statistical analysis codes will be made available in a public repository.

### 2.13. Monitoring

This multicenter trial will be conducted without a data-monitoring committee (DMC).

### 2.14. Harm’s Auditing

European medical device legislation and national implementing regulations apply.

All adverse events (AE) and serious adverse events (SAE) will be carefully recorded and evaluated (For specification see Table 3). Treatment complications are reported in the results of this study.

These events are complications related to the injury or treatment. They are categorized as resolved and long-term or unresolved complications. Among other things, the need for re-operation is also recorded here.

### 2.15. Auditing

AE’s will be immediately reported to Bioretec Ltd. and the study coordinator. SAE’s will be additionally reported to the local competent authorities.

### 2.16. Follow-Up Examination

Each study patient will undergo standardized examinations at defined time points preoperatively, immediately postoperatively, and during outpatient follow-up visits. An overview of the individual clinical parameters that should be collected at each visit is provided in Table 2.

### 2.17. Statistical Analysis

All patients with a follow-up of more than one year will be included in the primary analysis of the refracture rate. By using the approximation to normal large sample, the 95% confidence interval will be achieved.

In addition, the general linear mixed model (GLMM) will be used to estimate the refracture rate with center as a random factor. Within the GLMM model, the difference in re-fracture rate depending on the following variables will be explored:

- Fracture type determined by X-ray.
- Age.
- Gender.
- BMI.
- Surgical technique.
- Immobilization time.
- Bony union formation.

Secondary performance parameters will be reported primarily using descriptive statistics. Safety variables will be reported using descriptive statistics.

The potential dropouts for any reason will be recognized and reported, despite the lack of one-year follow-up data, whilst no comparisons of the main or secondary variables at endpoint will be available.

### 2.18. Research Ethics Approval

The study must be both approved and monitored by the local institutional review board (IRB) at each study site. All IRBs involved comply to the ethical principles of the Declaration of Helsinki for Medical Research Involving Human Subjects. This study has been registered with ClinicalTrials.gov on 15 April 2021(NCT04846543).

### 2.19. Protocol Amendments

All modifications of the study protocol have to be approved by the local Ethical Committee and will be communicated by updating the trial registry (ClinicalTrials.gov, accessed on 16 May 2022)

## 3. Operative Technique and Preliminary Results

Implantation of Acitva IM Nail is similar to the ESIN technique (Figure 2). After preparing an entry portal to the cortical bone with a bone awl or a drill bit, a steel dilatator is inserted into the medullary cavity and reduction is performed. In cases of dual-bone fractures, both bones are reduced and dilatators are inserted. Then, the steel dilatators are replaced by the biodegradable implants—one by one. As the PLGA implant should not be twisted or bent, the Activa IM Nail has to slide into the medullary canal smoothly. If the implant gets stuck, we recommend removing the implant and using the dilatator again. After insertion, the implant must be cut and smoothened at least to the cortical level to prevent soft-tissue irritation. Post operatively, cast immobilization is necessary, due to the lower stability of PLGA compared to titanium.

### 3.1. Preliminary Results (Pilot Study)

As of April 2022, 76 patients were enrolled as per study protocol; there were 31 girls (40.8%) and 45 boys (59.2%). The mean age at the time of inclusion was 8.9 years (±2.4 years). The left side was involved in 36 cases (47.4%), and the right side was involved in 40 (52.6%). A total of 23 fractures were in those aged 3 to 7 (30.3%), 29 in those aged 8 to 10 (38.2%), and 24 in those aged 11 to 13 (31.6%).

The mean time from trauma to treatment was 44.1 ± 49.8 h (range, 2.7–261.0). The mean operation time was 58.9 ± 22.9 min (range, 15–119 min); 35 fractures (46.1%) were treated within the first 24 h of trauma, and 41 fractures (53.9%) were treated after 24 h of initial trauma.

The mean duration of hospitalization was 1.6 ± 0.8 days (range, 1–3).

All patients were treated according to the treatment protocol (Figure 2). The mean follow-up time was 8.9 ± 5.1 months (range, 0.2–18.6); 41 out of 76 patients (54.0%) completed their 8-month or longer follow-up period and were evaluated by visits or were reached by phone. Overall, 65 patients had 3 months’ follow up (85.5%); the remaining 11 patients had surgery less than three months before the writing of the present article.

Fracture healing took place without any problems in all cases until the follow-up visit at twelve weeks. Three out of the seventy-six patients (3.9%) had secondary displacement less than two weeks after initial surgery even though an above the elbow cast was applied. There was no indication for revision surgery in these cases. All patients resumed to daily life and sport activities (see Table 4).

#### 3.1.1. Complications

In one case, a superficial wound infection was revised, and the surgeon found a fragment of the PLGA implant subcutaneously. In another case, a foreign body at the fracture site was detected after cast removal. Revision surgery was performed and a piece of the implant was removed. Further investigation is under way to analyze these two cases.

A lesion of the ramus dorsalis of the radial nerve—a well-described complication regarding the approach to the distal radius—was detected postoperatively in one case.

None of the patients developed a compartment syndrome or a Volkmann’s ischemic contracture.

#### 3.1.2. Refracture

Up to now, one refracture has occurred in one child falling from a height of about one meter 7 months after index surgery (1/76; 1.3%). The radiographs at 5 months postsurgery showed complete healing of the fracture. The refracture occurred at the same site as the initial injury. The second fracture was minimally displaced and was treated with long-arm cast immobilization; surgery was not necessary. According to Amerstorfer’s suggestion [11,24], 41 out of 76 patients (54.0%) had an 8-month or longer follow-up. In this subgroup of patients, refracture rate was 2.4% (one of 41), which is higher than calculated for the entire study group.

## 4. Discussion

### 4.1. Potential Impact and Significance of the Multicenter Trial

The overall objective of the present trial, and of its preliminary results (pilot study), is focused on the efficacy and safety of the Activa IM-Nail™ and the surgical procedure. Within the framework of a PMFC study, these parameters will be examined. PMCF studies are part of the necessary postmarketing surveillance of medical devices. The importance of postmarketing surveillance has been strengthened under the current MDR (medical device regulation) in terms of patient safety. These data are extremely important, especially for pediatric patients as a population requiring special protection.

The special aim of the pilot study is to preliminarily assess the refracture rate and to determine the subjective benefits in patients with forearm shaft fractures managed by bioresorbable hardware. A disadvantage of using the Activa IM-Nail™ is that patients have to be immobilized with plaster of Paris, which is not always necessary for patients treated with ESIN. This translates in a higher number of follow-up visits for patients treated by the Activa IM-Nail™ compared to those managed by ESIN.

The operative stabilization of forearm shaft fractures has increased with the introduction of ESIN. This can probably be explained by the less invasive procedure. ESIN has the advantage of smaller incisions, shorter operative time and less blood loss compared to open techniques such as plate fixation. Moreover, the increasing functional demands of the patients can represent an additional factor influencing the surgical indications. Full range of motion with unrestricted pronation and supination is the main goal of the treatment, even though reduced forearm motion can be compensated for by the shoulder.

Surgery is not free of complication. From the patient’s point of view, protruding or moving nails, skin irritation or pseudo-bursitis are considered to be less significant, though they can limit daily life activities; other serious complications such as infections are observed less often.

One of the well-described complications in diaphyseal forearm shaft fractures is refracture, which can occur after both conservative treatment and surgical stabilization.

In the publication of Korhonen 2018 [10], there was an increased rate of refractures (2/19 patients; 10.5%) when using resorbable nails in comparison to titanium nails in childhood, which would be a significant disadvantage of this treatment. However, it must be noted critically that the number of patients examined (*n* = 20) was too small to be able to make clear statements about the procedure. Our preliminary results suggest the refracture rate is lower than reported by Korhonen et al. 2018 [10].

Resorbable implants have multiple advantages for the patient. In particular, complications related to nail length with skin perforation or pseudo-bursitis can be significantly reduced due to the ability to shorten the nail to the level of the bone surface. Since no material removal is necessary, there is no need for an additional operation with hospitalization and a second general anesthesia, which may cause pain and anxiety for the patient, and stress and absence from work for the patient’s legal guardian, as well as increased costs in the health care system.

The use of resorbable implants represents an innovative technique. However, our results should be intended as preliminary, and the analysis of the whole cohort will provide more definitive data. Final data will represent the largest study population on the subject and will bring new insights about the indications, advantages and potential complications of the reported technique.

### 4.2. Strengths and Limitation of the Trial

This trial has potential limitations. The trial has only one interventional arm and there is no control group. For answering the primary research question (refracture) there is enough literature reporting refracture rate in children with forearm fracture managed by titanium ESIN. Therefore, the decision was made to compare the study group to patients from the existing literature. Lastly, this is an ongoing study, and preliminary results of the pilot study, including refracture rate, may vary once all patients are enrolled.

### 4.3. Expectations

In the context of the present multicenter PMCF study to evaluate safety and efficacy, our hypothesis is that there is no difference in the refracture rate between the patients treated by the bioabsorbable Activa IM-Nail™ and children managed by ESIN gathered from the available literature. Preliminary results of the pilot study support this hypothesis.

### 4.4. Trial Status

This trial is ongoing. Patient recruitment began in September 2020 at Péterfy Hospital, Hungary; in March 2021, at the study site in Austria; and in May 2021 at Pécs University Hospital. The study site at Hvidovre Hospital in Denmark received a positive vote from the Ethical Committee in July 2021 and started recruiting in the same month. The study site at Lübeck, Germany has a positive vote from the Ethical Committee and started recruiting in November 2021. The study site at Lille University Center-Jeanne de Flandre Hospital, France received a positive vote from the Ethical Committee in December 2021. At all other study sites, approvals are in process, including Ethical Committees’ decisions, and, therefore, recruitment has not started yet.

## 5. Conclusions

The multicenter postmarket clinical follow-up study will investigate the biodegradable intramedullary nailing of forearm fractures in pediatric patients in a prospective setting. In particular, the results should help to clarify the question of the refracture rate as a parameter of the effectiveness of Activa IM-Nails™ in forearm fractures in children. Furthermore, the impact of this procedure on the national economy is to be determined by means of a health technology assessment.

## Figures and Tables

**Table 1:** Study sites.

Country	Facility	Department	Adress	Facility Contact	Site Recruitment Status	Contact
Austria	Landesklinikum Baden/Mödling	Department of Orthopedics and Trauma	Sr. Maria Restitutagasse 12, 2340 Mödling	Christoph Röder	Recruiting	christoph.roeder@moedling.lknoe.at +43-2236-90040
Denmark	Hvidovre Hospital	Department of Orthopedics	Ketttegaard alle 30, 2650 Hvidovre	Louise Klingenberg	Recruiting	louise.klingenberg.03@regionh.dk +45-40884943
France	Jeanne de Flandre Hospital	Department of Pediatric Orthopedic Surgery	Avenue Eugene Avinée, 59000 LILLE	Federico Canavese	Recruiting	federico.canavese@chru-lille.fr +33-3-20446867
Germany	University Medical Center Schleswig-Holstein, Campus Lübeck	Department of Pediatric Surgery	Ratzeburger Allee 160, 23538 Lübeck	Ludger Tüshaus	Recruiting	ludger.tueshaus@uksh.de +49-451-50042611
Germany	Universitätsmedizin Mainz	Department of Orthopedics and Trauma Surgery	Langenbeckstraße 1 55131 Mainz	Erol Gercek	Not yet Recruiting	erol.gercek@web.de +49-6131-177292
Hungary	Dr. Manninger Jenő Baleseti Közpon	Department of Pediatric Trauma Surgery	1081 Fiumei út 17 Budapest	Marcell Varga	Recruiting	drvmarcell@gmail.com +36-70-9323027
Hungary	Pécs University Hospital	Department of Pediatrics	Jozsef Attila u. 7, 7623 Pécs	Gergo Jozsa	Recruiting	dr.jozsa.gergo@gmail.com +36-72-535900
Portugal	Hospital Pediátrico-CHUC, EPE	Department of Pediatric Orthopaedics	Av Afonso Romão 3000-602 Coimbra	Cristina Alves	Not yet Recruiting	6443@chuc.min-saude.pt +351-239-480355
Switzerland	Centre Hospitalier Universitaire Vaudois	Service de Chirurgie de l’enfant et de l’adolescent-SCEA	BH-11 Rue du Bugnon CH-1010 Lausanne	Nicolas Lutz	Not yet Recruiting	nicolas.lutz@chuv.ch +41-21-3148538

**Table 2:** Schedule of enrolment in the trial, intervention and follow up.

Time Point	Prior to Inclusion	During Surgery	2 Weeks after Surgery	4 Weeks	12 Weeks	1 Year	2 Years (Optional)
**Eligibility**	x						
**Informed consent**	x						
**Type of implant**		x					
**Radiological evaluation**		x	x	x	x		
**MRI scan (optional)**						x	x
**Duration of x-ray**		x					
**Time of operation**		x					
**Length of hospital stay**			x				
**Pain**			x	x	x	x	x
**ROM of elbow and wrist**			x	x	x	x	x
**Complications**		x	x	x	x	x	x
**(Serious) adverse events**		x	x	x	x	x	x
**Cost-effectiveness**						x	

**Table 3:** Adverse event (AE) and serious adverse event (SAE) specification.

AE	SAE
Intra-operative complications related to fixation hardware	Device malfunction
Need for secondary reduction due to fracture instability	Hospitalization
Mechanical implant failure	Medically important events
Incidence of infection	Accidental exposure
Osteolysis	Life-threatening events

**Table 4:** Patients’ characteristics and preliminary results (pilot study).

	All Patients	Patients > 8 Months Follow Up	Patients > 12 Months Follow Up
**Number of patients (*n*)**	76 (100%)	41 (54.0%)	16 (21.0%)
**Age (mean; sd; range) (years)**	8.87; ±2.41; 4–12	8.6; ±2.6; 4–12	8.9; ±2.1; 5–12
**Age group (3–7 years) (*n*)**	23 (30.3%)	16 (39.0%)	5 (31.3%)
**Age group (8–10 years) (*n*)**	29 (38.2%)	14 (34.2%)	8 (50.0%)
**Age group (11–13 years) (*n*)**	24 (31.6%)	11 (26.8%)	3 (18.8%)
**Female (*n*)**	31 (40.8%)	18 (43.9%)	8 (50.0%)
**Male (*n*)**	45 (59.2%)	23 (56.1%)	8 (50.0%)
**Left (*n*)**	36 (47.4%)	23 (56.1%)	9 (56.2%)
**Right (*n*)**	40 (52.6%)	18 (43.9%)	7 (43.8%)
**Follow up (mean; sd; range) (months)**	8.9; ±5.1; 0.2–18.6	12.7; ±3.3; 8.2–18.6	16.4; ±2.2; 12.1–18.6
**Time from trauma to treatment** **(mean; sd; range) (hours)**	44.1; ±49.8; 2.7–261.0	31.3; ±32.5; 2.7–170.3	26.0; ±38.1; 2.7–170.3
**Number of patients treated** **within 24 h**	35 (46.1%)	23 (56.1%)	12 (75.0%)
**Operation time** **(incision to wound closure)** **(mean; sd; range) (min)**	58.9; ±22.9; 15–119	57.2; ± 21.4; 20–105	63.3; ±20.4; 35–105
**Hospital stay (mean; sd; range) (days)**	1.6; ±0.8; 1–3	1.8; ±0.8; 1–3	2.4; ±0.6; 1–3
**Refracture rate (*n*)**	1 (1.3%)	1 (2.4%)	1 (6.2%)

## Data Availability

Principal investigators (PI) will control any use of trial data. At the time of publication, the final anonymized datasets and statistical analysis codes of the study results will be made accessible in a public database (e.g., Open Science Framework, osf.io).

## Appendix A

**Table A1:** List of Institutional Review Boards and Ethics Committees involved.

Study Site	Review Board/Ethics Committee	Approval Number	Date of Approval (mm-dd-yyyy)
Austria Baden/Mödling	Niederösterreichische Ethikkommission	GS4-EK-3/165-2020	11-09-2020
Germany	Ethik-Kommission der Universität Lübeck	21-258	08-05-2021
Hungary Pecs	PTE-KK Regionalis és Intézményi Kutatás—Etikai Bizottság	8737-PTE2021	23-04-2021
Hungary Peterfy	Péterfy Kórház Rendelőintézet, Országos Traumatológiai Intézet, Intézményi Kutatásetikai Bizottság	4/2020	11-05-2020
Denmark	De videnskabsetiske komiteer	H-21011210	07-07-2021
France	Comité de Protection des Personnes Est IV (CPP Est IV)	CNRIPH 21.02.01.44305	20-12-2021
